# Virologic outcomes of switching to boosted darunavir plus dolutegravir with respect to history of drug resistance

**DOI:** 10.1186/s12981-021-00384-6

**Published:** 2021-09-08

**Authors:** Eva Wolf, Christoph Boesecke, Annamaria Balogh, Helen Bidner, Christiane Cordes, Hans Heiken, Ivanka Krznaric, Tim Kümmerle, Hans-Jürgen Stellbrink, Jochen Schneider, Christoph D. Spinner, the DUALIS Study Group

**Affiliations:** 1 MUC Research, Clinical Research Organization (CRO), Waltherstr. 32, 80337 Munich, Germany; 2 Bonn University Hospital, Venusberg-Campus 1, 53127 Bonn, Germany; 3 Munich Study Center, School of Medicine, Technical University of Munich, Ismaninger Str. 22, 81675 Munich, Germany; 4 Private Practice, Warschauer Str. 33, 10243 Berlin, Germany; 5 Private Practice Georgstrasse, Georgstr. 46, 30159 Hannover, Germany; 6 Private Practice, Zentrum Für Infektiologie Prenzlauer Berg, Berlin, Germany; 7 Private Practice Am Ebertplatz, Ebertplatz 1, 50668 Köln, Germany; 8 ICH Study Center, Grindelallee 35, 20146 Hamburg, Germany; 9 School of Medicine, University Hospital Rechts Der Isar, Technical University of Munich, Ismaninger Str. 22, 81675 Munich, Germany

**Keywords:** Dolutegravir, Darunavir, HIV drug resistance, Integrase inhibitors, Protease inhibitors

## Abstract

**Objective:**

The DUALIS study showed that switching to boosted darunavir (bDRV) plus dolutegravir (DTG; 2DR) was non-inferior to continuous bDRV plus 2 nucleoside/nucleotide reverse-transcriptase inhibitors (NRTIs; 3DR) in treatment-experienced virologically suppressed people living with HIV (PLWH). We analyzed virologic outcomes with respect to treatment history and HIV drug resistance.

**Design:**

Post hoc analysis of a randomized trial.

**Methods:**

Main inclusion criteria were an HIV RNA level < 50 copies/mL for ≥ 24 weeks and no resistance to integrase strand transfer inhibitors or bDRV. Resistance-associated mutations (RAMs) were interpreted using the Stanford HIVdb mutation list. Outcomes measures were 48-week virologic response (HIV RNA < 50 copies/mL, FDA snapshot) and HIV RNA ≥ 50 copies/mL (including discontinuation due to a lack of efficacy or reasons other than adverse events and HIV RNA ≥ 50 copies/mL, referred to as snapshot non-response).

**Results:**

The analysis population included 263 patients (2DR: 131, 3DR: 132): 90.1% males; median age, 48 years; CD4 + T-cell nadir < 200/µl, 47.0%; ≥ 2 treatment changes, 27.4%; NRTI, non-NRTI (NNRTI), and major protease inhibitor (PI) RAMs in 9.5%, 14.4%, and 3.4%, respectively. In patients with RAMs in the 2DR and 3DR groups, virologic response rates were 87.8% and 96.0%, respectively; the corresponding rates in those without RAMs were 85.7% and 81.8%. RAMs were unrelated to virologic non-response in either group. No treatment-emergent RAMs were observed.

**Conclusions:**

DTG + bDRV is an effective treatment option without the risk of treatment-emergent resistance for PLWH on suppressive first- or further-line treatment with or without evidence of pre-existing NRTI, NNRTI, or PI RAMs.

*Trial registration*: EUDRA-CT Number 2015-000360-34; registered 07 April 2015; https://www.clinicaltrialsregister.eu/ctr-search/trial/2015-000360-34/DE.

## Introduction

Advances in the potency and resistance barrier of antiretroviral drugs for HIV infection, as well as evidence from recent randomized clinical trials (RCTs) in people living with HIV (PLWH), support the use of dual therapy including a boosted protease inhibitor (PI) or a second-generation integrase strand transfer inhibitor (INSTI) in specific patient populations such as PLWH on suppressive antiretroviral therapy (ART) without a history of virologic failure or — at least for some combinations — treatment-naïve PLWH [1–4]. Both the 2^nd^ generation INSTI dolutegravir (DTG) and the PI darunavir (DRV, boosted with ritonavir or cobicistat) have been shown to be potent antiretroviral drugs with a high resistance barrier in treatment-naïve and treatment-experienced PLWH [5–8]. The DUALIS study (Eudra-CT 2015-000360-34), a phase IIIb, open-label 48-week RCT in virologically suppressed PLWH, demonstrated that switching to a 2-drug regimen (2DR) consisting of boosted DRV (bDRV) plus DTG was non-inferior to continuing a 3-drug regimen (3DR) including bDRV plus two nucleoside/nucleotide reverse-transcriptase inhibitors (NRTIs). The week-48 virologic response rates based on Food and Drug Administration snapshot analysis (HIV RNA < 50 copies/mL, the primary endpoint) were 86% (2DR) and 88% (3DR) in the exposed intention-to-treat (ITTe) analysis population. The difference between the 2DR and 3DR study arms was − 1.6% [95.5% confidence interval (CI), based on an alpha level adjusted for interim analysis: − 9.9 to + 6.7%]. The rates of snapshot virologic non-response were 3.8% (2DR) and 5.3% (3DR), with a difference of − 1.4% (95% CI − 6.5 to + 3.6%) [9]. In this post hoc analysis of the DUALIS study, historic genotypic resistance patterns were assessed and primary and secondary virologic outcomes in the 2DR and 3DR study arms were evaluated with respect to treatment history and HIV drug resistance.

## Methods

The study population and statistical methods of the DUALIS study have been previously described [9]. The inclusion criteria were an HIV RNA level of < 50 copies/mL for at least 24 weeks before screening (one viral blip of ≤ 200 HIV RNA copies/mL was accepted), bDRV-based ART for at least 4 weeks, and no history or presence of genotypic or phenotypic drug resistance to INSTIs or bDRV. A history of HIV treatment failure was not exclusionary. The resistance-associated mutations (RAMs) of interest were based on the Stanford HIVdb mutation list (HIVdb version 8.9-1 as of 2019-10-25) [10]; PI RAMs were classified as major or minor mutations. Primary and secondary virologic outcomes were stratified by treatment line and the presence of NRTI, NNRTI, and minor and/or major PI RAMs. Virologic outcomes as per FDA snapshot algorithm included an HIV RNA level < 50 copies/mL in the 48-week visit window (the primary endpoint, referred to as virologic response) and an HIV RNA level ≥ 50 copies/mL (including HIV RNA level ≥ 50 copies/mL in the 48-week visit window *OR* study/study drug discontinuation due to: (i) a lack of efficacy, or (ii) reasons other than adverse events or death, with the last HIV RNA level being ≥ 50 copies/mL; hereinafter referred to as snapshot virologic non-response). Since this is a post hoc analysis, only descriptive statistics have been applied (without statistical testing).

## Results

### Study population, treatment history, and HIV drug resistance

The ITTe analysis population of the DUALIS study included 263 patients (2DR, n = 131; 3DR, n = 132): 90.1% were of male sex; median age, 48 years; Centers for Disease Control category C, 29.7% of patients; and CD4 + T-cell nadir < 200/μL, 47.0% of patients. The median duration of ART was 5.3 years; 27.4% of the patients had a history of at least two ART changes, including ≥ 2 NRTI and/or ≥ 2 PI changes in 20.9% and 11.0% of the patients, respectively. Prior INSTI use was documented in 8.4% of the patients (n = 22; 3/22 with exposure to 2 INSTIs; raltegravir, n = 16; elvitegravir/cobicistat, n = 5; dolutegravir, n = 4).

Former or current reports of genotypic resistance were available for 63.5% of the patients (167/263). NRTI, NNRTI, and/or (major or minor) PI RAMs were observed in 9.5%, 14.4%, and 30.0% of the ITTe set, respectively. Major PI RAMs were documented in 3.4% of the patients. ART-related history and the prevalence of RAMs stratified by the study arms are shown in Table 1.

**Table 1:** ART-related history and the prevalence of resistance-associated mutations (RAMs)

	2DR (N = 131)	3DR (N = 132)
Duration of ART [years]; median (IQR)	5.1 (3.5–8.3)	5.5 (3.4–8.4)
Prior INSTI use^a^; n (%)	9 (6.9)	13 (9.8)
History of ≥ 2 NRTI changes; n (%)	22 (16.8)	33 (25.0)
History of ≥ 2 PI changes; n (%)	17 (13.0)	12 (9.1)
History of ≥ 2 ART changes; n (%)	27 (20.6)	45 (34.1)
Any major/minor RAM; n (%)	49 (37.4)	50 (37.9)
NRTI RAMs; n (%)	13 (9.9)	12 (9.1)
NNRTI RAMs; n (%)	20 (15.3)	18 (13.6)
Any major/minor PI RAM; n (%)	38 (29.0)	41 (31.1)
PI RAM, major (± minor); n (%)	5 (3.8)	4 (3.0)
PI RAM, minor; n (%)	33 (25.2)	37 (28.0)

RAMs prior to baseline (in one or several cases): NRTI RAMs (M41I, M41L, A62V, D67N, T69N, T69S, K70R, V75I, F77L, Y115F, F116Y, M184I, Q151M, M184V, T215C, T215D, T215E, T215F, T215I, T215S, T215Y, K219E, K219Q); NNRTI RAMs (V90I, K101E, K101P, K101Q, K103N, K103R, V106I, V108I, E138A, E138K, V179D, V179E, V179I, V179T, Y181C, Y181V, Y188L, V189I, G190A, M230I); major PI RAMs (based on HIVdb version 8.9-1 as of 2019-10-25) [10]; D30N, V82F, V82S, V82T, N88S, L90M); and minor PI RAMs (L10I, L10M, L10V, K20I, K20M, K20R, K20T, L33F, L33V, E35D, M36I, M36L, R41K, K43T, I62V, L63P, H69K, A71I, A71L, A71T, A71V, T74S, V77I, N88D)

^a^Prior INSTI use, n: raltegravir, 16; elvitegravir/cobicistat, 5; dolutegravir, 4; (n = 3 with prior use of two INSTIs)

### Viral response (HIV RNA < 50 copies/ml) at week 48 with respect to treatment history and HIV drug resistance

**Fig. 1:**
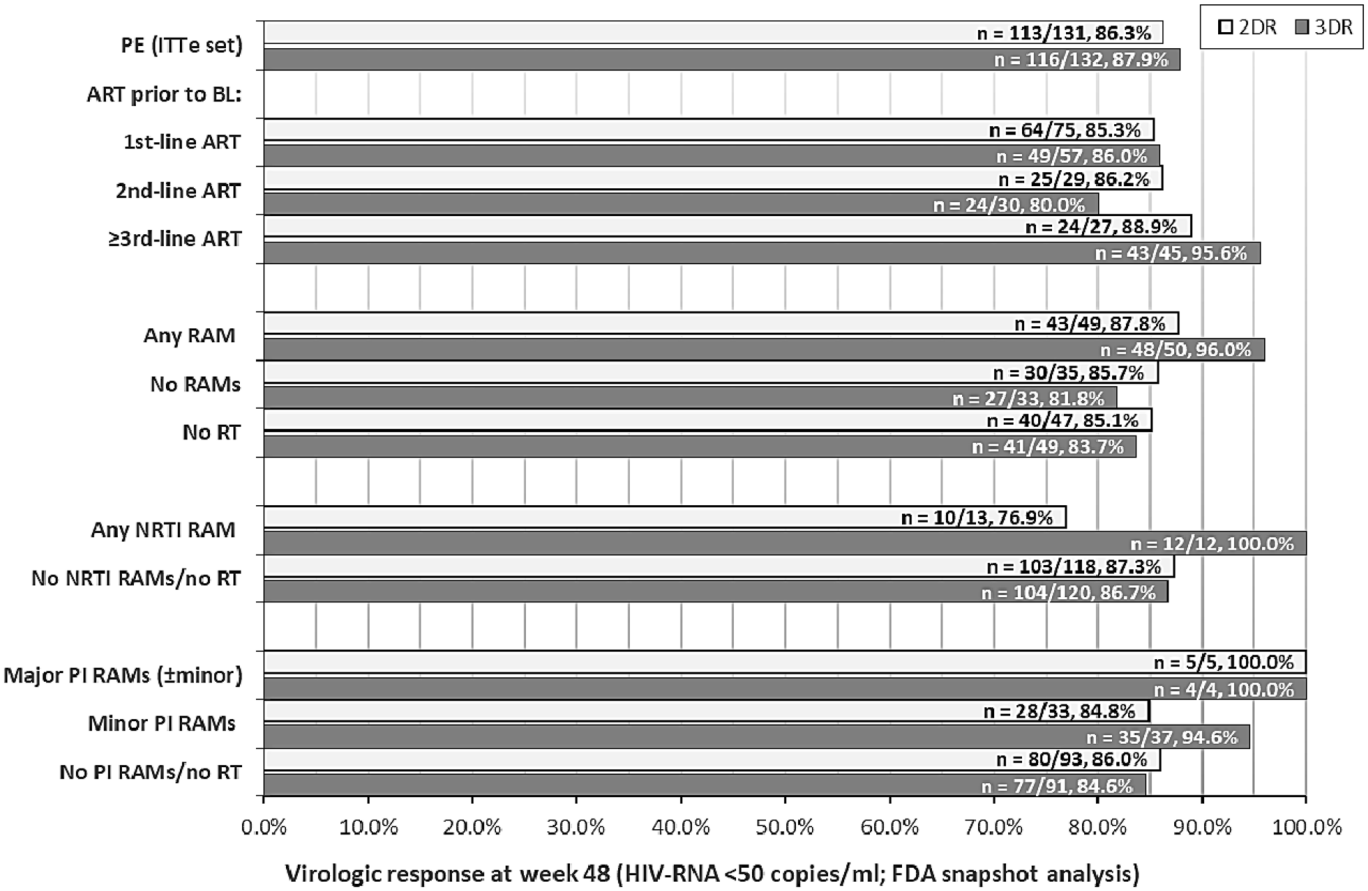
Primary endpoint analyses. Primary endpoint (PE) analyses in the exposed intention-to-treat (ITTe) analysis population, overall and in the subgroups based on treatment experience and history/presence of resistance-associated mutation (RAMs). *RT* resistance testing, *NRTI* nucleoside/nucleotide reverse transcriptase inhibitor, *PI* protease inhibitor

Primary endpoint analyses with respect to treatment history and the presence of RAMs are shown in Fig. 1. Comparison of the patients with and without documented RAMs revealed response rates of 87.8% and 85.7%, respectively, in the 2DR group. The corresponding rates in the 3DR group were 96.0% and 81.8%.

### Snapshot virologic non-response (HIV RNA ≥ 50 copies/ml) at week 48 with respect to treatment history and HIV drug resistance

For patients on first-line, 2^nd^-line or ≥ 3^rd^-line ART prior to baseline, snapshot virologic non-response rates were 1.3% (one out of 74), 6.9% (two out of 29), and 7.4% (two out of 27) in the 2DR group, and 5.3% (three out of 57), 10.0% (three out of 30), and 2.2% (one out of 45) in the 3DR group, respectively. Of the study participants with major or minor RAMs before baseline, virologic non-response was observed in 2.0% in each study arm (one out of 49 and one out of 50 patients in the 2DR and 3DR arms, respectively). In the 2DR subgroup, virologic non-response was observed in a case with minor PI mutations/polymorphisms (M36I and L63P) and viral mutations associated with NRTI resistance (A62V, T69S, V75I, F77L, F116Y, and Q151M) before baseline. In the 3DR subgroup, virologic non-response was observed in a patient with only a minor PI mutation (A71T) prior to baseline.

Grouping patients without documented RAMs before baseline and patients without available historic resistance test data, virologic non-response rates were 4.9% in the 2DR group (four out of 82 patients) and 7.3% in the 3DR group (six out of 82). In patients without RAMs, virologic non-response was observed in 7.4% of study participants (five out of 68; 2DR: 2.9%, one out of 35; 3DR: 12.1%, four out of 33). In patients without resistance test, non-response was observed in 5.2% of study participants (five out of 96; 2DR: 6.4%, three out of 47; 3DR: 4.1%, two out of 49). During the follow-up, no emergence of RAMs was observed in either study arm. Of the nine patients with documented major PI RAMs, no patient in either group experienced virologic non-response. The same was true for the 12 patients with NRTI RAMs in the 3DR study arm.

## Discussion

In the DUALIS study, dual therapy with DTG plus bDRV was an effective treatment option with no treatment-emergent resistance in PLWH on suppressive first- or further-line ART with or without evidence of pre-existing NRTI, NNRTI, or PI RAMs. Neither the presence of major or minor PI RAMs (not conferring phenotypic resistance to DRV based on the interpretation of resistance patterns) nor that of NRTI or NNRTI RAMs (assessed based on available historic genotypic resistance reports) affected the maintenance of virologic response after switching to DTG plus bDRV over 48 weeks. However, a limitation of this post hoc analysis of the DUALIS study is the only low to moderate prevalence of relevant RAMs. Prior to baseline, NRTI, NNRTI, or major PI RAMs were observed in 9.5%, 14.4%, and 3.4% of the analysis population, respectively. Moreover, the information on primary resistance and/or history of emerging resistance mutations pertaining to previous antiretroviral regimens was not available for approximately one-third of the study population. Of note, post hoc measurement of archived resistance mutations in proviral DNA was not foreseen in the study. The reason for missing historic resistance information could have been that primary resistance had not been evaluated before treatment initiation or resistance testing had not been available or feasible at the time of previous virologic failure in some cases (which was not an exclusion criterion of the study). However, these probably missed opportunities for resistance testing might indicate an underestimated prevalence of RAMs, namely that even more mutations may have been present in the study population than documented. Of note, transmitted HIV drug resistance in Germany has been reported to be relatively stable at approximately 10% [11].

Real-word data are in line with the results of this study. Several cohort studies evaluating bDRV plus DTG in PLWH with extended treatment experience and an even higher prevalence of NRTI, NNRTI, and major PI RAMs than in the DUALIS study confirmed a high virologic effectiveness and a high resistance barrier of the dual regimen [12–15]. In a small retrospective cohort study, 49 of 50 PLWH (98%) maintained viral suppression on bDRV and DTG for a median of 25 months without the emergence of new RAMs [14]. In a prospective cohort study evaluating treatment simplification to once daily bDRV plus DTG in 51 PLWH, the viral response rate at week 48 was 90% without any case of virologic failure [15]. In another observational study, 130 multidrug-experienced HIV-infected subjects from 11 Italian centers who had been switched to bDRV plus DTG between 2014 and 2015 were followed up for at least 96 weeks. The reasons for switching were treatment simplification, virologic failure, and toxicity in 45%, 30%, and 17% of patients, respectively. At baseline, 91% had documented resistance to one or several antiretroviral drug classes; 40% had uncontrolled viral replication. At week 96, only two patients had a plasma viral load of ≥ 50 HIV RNA copies/mL, and 95% had < 50 HIV RNA copies/mL, demonstrating that the two-drug regimen consisting of bDRV plus DTG may even be a rescue treatment option in case of virologic failure [16]. Of note, pharmacokinetic data from the PK I and PK II DUALIS sub-studies and a study in healthy volunteers showed adequate plasma levels of DRV and DTG, supporting the safety of the combination without dose adjustments [17–19]. Moreover, single-point measurements as well as full pharmacokinetic profiling nested in the prospective Swiss HIV Cohort Study showed that even advanced age (≥ 65 years) only minimally affected DTG exposure and did not affect boosted DRV exposure to a clinically significant extent [20]. In view of the favorable pharmacokinetic and resistance profiles of bDRV plus DTG and given the experience with this combination from real-world settings, the DUALIS study was the first controlled RCT addressing the evidence gap for this dual therapy in treatment-experienced PLWH. This post hoc analysis of the study confirmed the robustness of switching to bDRV plus DTG in virologically suppressed patients with or without a history of RAMs or without historic resistance tests, but without clinical evidence of resistance to INSTIs or DRV. In conclusion, virus suppression can be maintained in treatment-experienced PLWH switched to a fully active 2-drug regimen consisting of bDRV plus DTG without an increased risk of de novo evolution of drug resistance.

## Data Availability

The datasets generated and/or analysed during the current study are not publicly available but are available from the corresponding author on reasonable request.
